# Emerging Novel Combined CAR-T Cell Therapies

**DOI:** 10.3390/cancers14061403

**Published:** 2022-03-09

**Authors:** Anh Nguyen, Gary Johanning, Yihui Shi

**Affiliations:** 1 College of Graduate Studies, California Northstate University, Elk Grove, CA 95757, USA; anh.nguyen7553@cnsu.edu; 2 SunnyBay Biotech, Fremont, CA 94539, USA; garyj947@gmail.com; 3 College of Medicine, California Northstate University, Elk Grove, CA 95757, USA

**Keywords:** chimeric antigen receptor (CAR), T cell, immunotherapy, hematologic malignancies, solid tumor, target antigen, tumor-associated antigen (TAA), tumor-specific antigen (TSA), checkpoint inhibitor, combined therapy

## Abstract

**Simple Summary:**

As a result of FDA approval of CAR-T cell treatments in the last few years, this immunotherapy has provided further direction to precision medicine through its combination with other therapeutic approaches. In the past year, several review articles have been published focusing on advances in this fast-developing field, especially with respect to efforts to overcome hurdles associated with applying CAR-T cells in solid tumors. This review paper focuses on combining CAR-T cell therapy with small molecule drugs, up-to-date progress in CAR-T cell therapy research, and advances in combined CAR-T immunotherapy with other treatments targeting solid tumors.

**Abstract:**

Chimeric antigen receptors (CAR) T cells are T cells engineered to express membrane receptors with high specificity to recognize specific target antigens presented by cancer cells and are co-stimulated with intracellular signals to increase the T cell response. CAR-T cell therapy is emerging as a novel therapeutic approach to improve T cell specificity that will lead to advances in precision medicine. CAR-T cells have had impressive outcomes in hematological malignancies. However, there continue to be significant limitations of these therapeutic responses in targeting solid malignancies such as heterogeneous antigens in solid tumors, tumor immunosuppressive microenvironment, risk of on-target/off-tumor, infiltrating CAR-T cells, immunosuppressive checkpoint molecules, and cytokines. This review paper summarizes recent approaches and innovations through combination therapies of CAR-T cells and other immunotherapy or small molecule drugs to counter the above disadvantages to potentiate the activity of CAR-T cells.

## 1. Introduction

**Figure 1:**
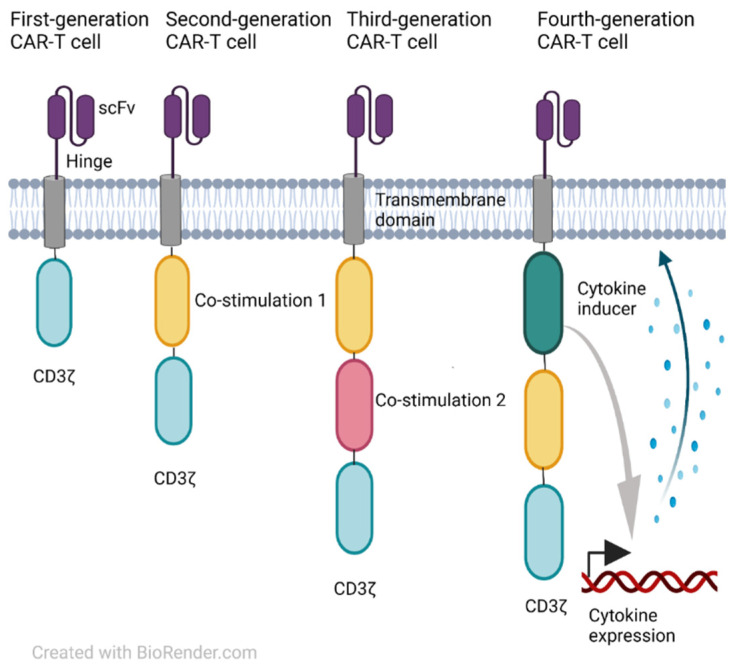
The general structure of different CAR generations.

CAR T-cell therapy entails the engineered modification of autologous T cells to robust T cells that can initiate anti-tumor reactivity of the target tumor cells. These therapeutics have produced meaningful clinical outcomes in the treatment of hematologic cancers, but have not produced comparable responses in solid malignancies. Notably, CAR-T therapy with the most investigated target thus far, CD19, is a promising therapy for hematologic cancer and has dramatically improved the treatment of lymphoid malignancies, predominantly diffuse large B-cell lymphoma (DLBCL) and acute lymphoblastic leukemia (ALL). Besides CD19, other common targets include B cell maturation antigen (BCMA), CD20, mesothelin (MSL), and PD-1/PD-L1 [1,2,3]. The CAR construct has become progressively more advanced over time. First-generation CARs have a single activatory domain, the CD3ζ intracellular signaling domain [4]. Second and third-generation CARs, alongside the CD3ζ domain, incorporate co-stimulatory domains, CD28 (second generation) or two or more co-stimulatory domains, CD27, 4-1BB, ICOS, or OX40 (third generation) [5,6,7]. Fourth-generation CARs contain a constitutive or inducible expression of co-receptors or soluble cytokines upon recognition by a specific antigen [8] (Figure 1). Despite significant progress in CAR constructs that further promote T-cell activation, there are limitations of CAR T-cell therapy that have encouraged scientists to shed light on future perspectives by exploring combined CAR-T cell therapy.

## 2. CAR-T Cells and Nanobodies

Previously, TCR-like antibody CAR-T cells with scFv recognizing the MHC/peptide complex have improved T cell activation and proliferation by targeting a variety of intracellular antigens of solid tumors [9]. Subsequent to this approach, TCR-like nanobody CAR-T cells have been developed to secrete small-sized nanobodies that serve as an antigen-binding domain. These constructs infiltrate tumor tissue and overcome tumor antigen escape. Some examples include nanobodies targeting TAG-72, HER2, or MUC1 [10,11]. Similar to TCR-like antibodies, nanobody CAR-T cells can trigger endogenous immune responses and overcome the absence of specific biomarkers on the surface of a range of tumor cells [9].

Nanobody enhanced immunoactivity resulting from the presence of an antigen-binding domain on CAR-T cells through its high affinity and stability has been applied in different generations of CARs. Nanobody second-generation CAR-T cells exhibited over 50% positive expression on the cell surface, thus secreting IL-2 and IFN-γ and boosting the cytotoxicity in VEGFR2 expressing cells [12]. Additionally, nanobodies serve as attractive modules for third- and fourth-generation CARs.

Bivalent nanobodies in bi-specific CAR-T cells significantly reduced tumor escape by simultaneously recognizing two targets, for example, CD20 plus either HER2, PD-L1, or EIIIB, on the cells with a resultant loss of target expression [13,14]. Most notably, De Munter et al. designed a TCR-like nanobody CAR-T therapeutic with two tandem TCR-like nanobodies bispecific to HER2 and CD20 to activate T cells and kill tumor cells [13]. Subsequently, other studies combined a TCR-like nanobody CAR-T with nanobodies targeting immune checkpoint inhibitors to address the tumor microenvironment to enhance the persistence of CAR-T cells and suppress tumor growth [15,16,17]. These approaches provide compelling options to improve the nanobody-based antigen binding domain in CARs that are directed to the MHC complex with the specificity of TCR and increased affinity to CARs [18].

## 3. CAR-T Cells and Immune Checkpoint Inhibitors

Over the past seven years, extensive research on the inhibition of immune checkpoints targeting PD-1 and PD-L1 has resulted in the rapid expansion of the class of checkpoint inhibitor drugs. More than 2000 clinical trials have been studied that focused on over 30 anti-PD-1 or anti-PD-L1 antibodies [19]. Among them, many drugs have been approved by the FDA due to their therapeutic advances. However, they fall into similar, if not identical, mechanisms of action, without further study to determine the superiority of one approach over others, suggesting that scientists consider advancing the future of oncologic drug development through combination treatments with CAR-T immunotherapy [19].

### 3.1. PD-1 Blockade with Different Immune Checkpoint Inhibitors

Inhibitors specific to immune response checkpoint ligands on tumor cells or receptors on T cells are designed to block the immune response to the tumor. CAR-T cell therapy incorporated with PD-1 blockade has demonstrated a synergistic effect in the treatment of hematological malignancies: for example, PD-1 blockade coupled with CD19 CAR-T cell therapy. Masked CAR is another strategy to overcome cytotoxicity in normal cells while showing tumor-killing activity on EFGR-positive human tumors. Researchers have designed EGFR-targeting CARs that lead to inactive PD-1 in the non-cancerous environment but are selectively activated through proteolytic cleavage by protease present in the tumor environment [20,21]. In clinical trials, different checkpoint inhibitors such as pembrolizumab and durvalumab can be combined with CAR-T cells for a synergistic effect (NCT03310619; NCT03630159, ClinicalTrials.gov). Equally important, a combination of HER2-targeted CAR-T cells with a blockade of PD-1 or GPC1 significantly enhanced tumor-killing activity in a mouse model, while anti-PD1 antibody or CAR-T cells alone showed partial inhibition of tumor growth [22,23,24]. Inhibition of immune checkpoint PD-1 also enhances the anti-tumor effectiveness of MSLN- or cMet-targeted CAR-T cells toward hepatocellular carcinoma [25,26]. CAR-T cells co-cultured with the target cells increased the secretion of IL-2 and IFN-γ, and the addition of the anti-PD1 antibody further elevated these two cytokines [24]. For the first time, combined PD-1 blockade, and CAR-T cell treatment achieved long-term therapeutic response and successfully treated a 37-year-old female patient with relapsed/refractory diffuse large B-cell lymphoma (R/R DLBCL) [27]. In another study, Adusumilli et al. demonstrated the potential applicability of MSLN-targeted CAR T-cell therapy in combination with the anti-PD-1 agent pembrolizumab in patients with malignant pleural disease for the first time. The overall response rate improved to 72% compared with current therapies [28], holding promise for combined CAR-T therapy in solid tumors, as there has only been one case report of a response to CAR-T therapy in a solid tumor prior to this study. The results also demonstrated that the anti-PD-1 agent was able to rejuvenate CAR-T cells and convert immunologically “cold” to “hot” tumors. Furthermore, blocking the PD-1 signal effectively overcame the exhaustion of CAR-T cells. Combined immunotherapy with CAR-T cells and PD-1 blockade agents should be further evaluated in patients with solid tumors [28]. Recently, anti-PD-L1 inhibitors combined with cyclophosphamide and oxaliplatin synergistically improved CAR-T cell-mediated tumor control and survival in breast and lung cancer patients. In this study, cyclophosphamide provided transient control of tumor growth, while oxaliplatin improved CAR-T cell infiltration [29]. Chong et al. also reported a successful combination of CD19-CAR-T cells and PD-1 blocking antibody after treating a patient with refractory diffuse large B-cell lymphoma. These combination therapies with CAR-T cells and inhibitory receptor blockade are thus emerging as a new strategy to overcome tumor escape and strengthen the CAR-T cells’ efficacy further [30].

However, CAR-T cell combination therapy with inhibitory checkpoint PD-1 blockade could present potential side effects, especially cytokine release syndrome (CRS). Checkpoint inhibition has notably shown one of the most significant adverse effects in CAR-T immunotherapies [31]. The most current CRS data from current CAR-T cell and blinatumomab studies in hematologic malignancies reported that CRS occurred in frequencies of up to 100% in CD19-targeted CAR T cell trials [32,33].

### 3.2. Armored CAR-T Cells Secreting Immune Checkpoint Inhibitors

The encouraging synergistic therapeutic effect of checkpoint inhibitors and CAR-T cells gives rise to the development of armored CAR-T cells that secrete immune checkpoint inhibitors to overcome immunosuppression within solid tumors, which possess physical and immune obstacles that limit CAR-T effectiveness [34]. Novel armored CAR-T therapies have been generated to overcome the immunosuppressive tumor microenvironment as a significant barrier to solid tumors. CAR-T cells can be modified to secrete antibodies to inhibit checkpoints (anti-PD-1/PD-L1/CTLA-4) for enhanced strength, effectiveness, and persistence of CAR-T therapy [14,22,35]. Suarez et al. created CAR-T cells that can secrete anti-PD-L1 antibodies to achieve a prolonged elimination of cancer cells instead of administering PD-1 blocking antibodies [36]. CAR-T cell therapy in the solid tumor effectively enhances the infiltration of immune cells with increased secretion of local anti-PD-L1 antibodies. Investigators have developed CAR-T cells with anti-PD-1 self-secreting or PD-1 silencing capabilities to block the negative regulatory pathway PD-1. PD-1-blocking CAR-T cells targeting EGFRvIII-expressing cells showed improved anti-tumor activity and prolonged survival in a mouse model of glioblastoma [20]. Rafiq et al. further developed CAR-T cells that secrete a PD-1 blocking scFv to enhance tumor suppression. An scFv-derived pro-antibody against PD-1 demonstrated augmented tumor-killing compared to the combinatory strategy of PD-1 disruption and CAR-T therapy [20,21,37,38]. Armored CAR-T cells carry a cytosolic transcription domain to facilitate the expression of immunogenic cytokines, receptors, or antibody-like proteins. Alongside CAR-T cells, these molecules collectively enhance T cell redirection to tumor and effector function in killing solid tumor cells [39]. Fang et al. designed CAR-T cells with an autocrine anti-PD-1 scFv module to promote the modulation of T cell function: CAR-T was only active after antigen engagement to control the secretion of anti-PD-1 antibodies at the tumor site. These investigators co-transfected a promoter construct upstream of the anti-PD-1 antibody gene and the CAR construct into T cells. These anti-PD-1 CAR-T cells had lower PD-1 expression, higher levels of T cell activation, and better anti-tumor activity, specifically against MSLN- and PD-L1-positive tumor cells both in vitro and in vivo [40].

### 3.3. TGF-β Receptor Blockade and Secretion of Pro-Inflammatory Cytokines

Tumor cells suppress the immune response at tumor sites through a strategy involving increased TGF-β-mediated repression of T-cell proliferation coupled with a Treg-mediated hostile microenvironment [41]. CAR-T cells designed to express a CD28-positive co-stimulatory domain reduced the inhibition caused by Treg cells and reversed the repression by TGF-β of CD4-positive and CD8-positive T cells [42]. Additionally, blocking the TGF-β receptor in CAR-T cells inhibited the release of immunosuppressive cytokines and increased tumor-associated T cells [41]. Chen et al. further improved the CAR construct by engineering CAR-T cells that secrete a bispecific trap protein co-targeting PD-1 and TGF-β for more potent anti-tumor immunity. The trap protein simultaneously blocks immune checkpoints and inhibits TGF-β-mediated differentiation of Tregs. In a mouse model, CAR-T cells with trap protein secretion were superior in tumor infiltration and anti-tumor efficacy than parental CAR-T cells and anti-PD-1 autocrine CAR-T cells, suggesting the potential to eradicate solid tumors and prevent tumor relapse [43]. Cadilha et al. were able to suppress Tregs by synergizing C-C chemokine receptor 8 (CCR8) and dominant-negative TGF-β receptor 2 (DNR) into CAR-T cell fusion. As CCR8 is known to direct Tregs to the tumor site, researchers applied CCR8 to redirect engineered T cells to the tumor site, while DNR shielded TGF-β for improved therapeutic efficacy. The introduction of CCR8 and DNR into CAR T cells provides more effective solid anti-tumor activity [44]. Furthermore, CAR-T cells engineered to enhance the release of the pro-inflammatory cytokines IL-2 and IL-12 prolonged T cell persistence and augmented the anti-tumor activity [45,46,47]. Furthermore, generated CAR-T cells secreting cytokine IL-2 maintained higher potency and persistence than normal CAR-T cells [48,49]. Another promising development area is the construction of CAR-T cells that initiate an endogenous immune response toward the tumor. Curran et al. developed an anti-CD19 CAR-T cell that expressed ligand CD154 for CD40, a co-stimulatory receptor on antigen-presenting cells. The authors demonstrated that CAR-T cells could increase the immunogenicity of CD40-positive cells and induce IL-2-secretion, thus increasing the potency of the CAR-T cells [50,51,52].

### 3.4. Switching the Original Signals of Negative Feedback Inhibition by CD28

Cytokines are released as part of the immune response and play a role in the immunosuppressive environment at tumor sites that promotes tumor progression. Certain cytokines upregulate the expression of PD-1 and PD-L1 to inhibit T cell activation and proliferation, leading to immunosuppression and negative feedback inhibition. Investigators modified a third-generation CAR to express PD-L1 and CD28 fusion receptors aimed at suppressing the inhibition with CD28. They fused the extracellular domain of PD-1 with the intracellular domain of CD28. Upon binding its extracellular ligand PD-L1, the fusion receptor transmitted an activation signal instead of an inhibitory signal with the CD28 cytoplasmic domain, hence a “switch”. These switch receptors improved cytokine release, proliferation, and cytotoxicity of CAR-T cells [53]. Furthermore, Liu et al. noted that these switch receptors increased the anti-tumor activity of CAR-T cells in solid tumors. The experimental data showed that the PD-1-CD28 receptor could more efficiently reduce tumor burden than the PD-1 blocking antibody alone [54,55,56].

## 4. CAR-T Cells and Small Molecules

Targeted therapies such as monoclonal antibodies and small-molecule inhibitors have improved CAR-T cell infiltration and specificity. Monoclonal antibodies target specific antigens found on the cell surface, whereas small-molecule drugs can translocate through the plasma membrane to interfere with the enzymatic activity and signaling pathways associated with tumor growth, survival, angiogenesis, and metastasis [57]. Monoclonal antibodies can either target the receptors overexpressed on tumor cells (such as HER2, EGFR, VEGFR, etc.) or target their ligands to neutralize those receptors on the cell surface (such as bevacizumab for VEGF).

The majority of small molecules target signal transduction mechanisms of proliferation and survival in many tumors; for example, tyrosine or serine/threonine kinases and the mitogen-activated protein kinase (MAPK) signaling cascade including RAS, RAF, MEK, and ERK. Kinase inhibitors can target several growth factor receptors with aberrant activation or deregulation in tumors such as EGFR, VEGFR, IGFR, and FGFR. VEGF signaling induces the suppression of CD8-positive T cell proliferation and reduces their cytotoxic activity [58]. Moreover, VEGF-A can induce the expression of PD-1 on tumor-infiltrating CD8-positive T cells, suggesting that CAR-T cells might benefit from VEGF-VEGFR pathway blockade [59].

Giordano-Attianese et al. designed CAR-T cells that can be switched off through its chemically disruptable heterodimer by administering a small-molecule drug. These switchable CAR-T cells provide a controllable means to improve the safety of CAR-T cell therapy and reduce the risk of toxicity while mounting similar anti-tumor killing activity as second-generation CAR-T cells [60]. Equally important, T cell exhaustion or fratricide due to the rapid expansion of targeted antigens is also of concern. Juillerat et al. eliminated non-specific activation with the benefit of preventing T-cell differentiation using a small molecule in a switch OFF fashion [61]. The SWIFF-CAR construct comprises the CAR followed by a protease target site, a protease, and a degradation moiety. The protease cleaved the degradation moiety from the CAR, exposing the antigen-targeting scFv at the T-cell surface (“ON” state). The presence of the protease inhibitor Asunaprevir inhibited the cleavage of the degradation moiety from the CAR by protease, leading to the degradation of the CAR by the T-cell proteolytic pathways, controlling CAR T-cell functions at the protein level [61].

Collectively, these results suggest that a systematic and integrated understanding of the molecular consequences of small molecules on CAR-T cells, tumor cells, and the microenvironment has been implemented for the optimal design of combined therapies to limit the risk of toxicities.

## 5. Novel Types of CAR T Cells Are Generated to Overcome the Lack of Ideal Tumor-Associated Antigens or Loss of Antigen in Solid Tumors

**Figure 2:**
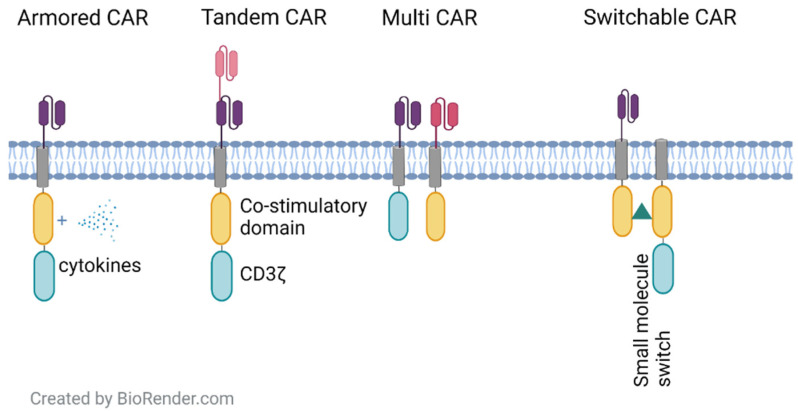
Various CAR constructs aimed at overcoming the loss of antigen and tumor heterogeneity.

These studies collectively suggest that novel CAR-T cell modifications and combinations are being rapidly introduced with the goal of enhancing the efficacy of CAR-T therapy or reducing unfavorable responses. These new CAR-T therapies can be partitioned into several major groups (Figure 2).

### 5.1. Multi-Target CAR-T Cells

Multi-target CAR-T cells address tumor antigen escape through multiple antigen-targeting CARs on a single CAR-T cell to overcome antigen loss or faded expression of a single antigen. Each unispecific CAR has a different individual binding domain. Multi-target CAR-T cells exert efficiency in the cytolysis of tumor tissues due to the high density of CAR expression on the surface of T cells. An example is multi-target CAR-T cells simultaneously expressing a receptor for the activation of T cells via CD3z and the antigen-MHC complex and another receptor such as CD28 and/or CD137 for a co-stimulatory signaling process [62]. Given heterogeneity and antigen escape variants, CAR-T cells need to target more than one antigen including multiple tumor-associated antigens (TAAs) simultaneously or other factors specific to the tumor environment of interest [63]. Yan et al. designed CD19/CD123 CAR-T cells that significantly reduce the rate of relapse and tumor burden and are superior in animal survival in the NSG mouse model of B-cell acute lymphoblastic leukemia (B-ALL). An in vitro study demonstrated that biCAR-T cells exhibited robust anti-tumor ability by ablating over 90% of target cells expressing both CD19 and CD123 [64]. BiCAR-T cells simultaneously expressing HER2 and IL13Rα2 CAR molecules showed higher efficiency in removing glioblastoma tumor cells and lower antigen escape variants than CAR-T cells targeting HER2 alone or pooled CAR-T cells in mouse xenograft models [65]. In in vitro breast cancer, the proliferation and anti-tumor efficacy of biCAR-T cells depend on combinational targeting of HER2 and MUC1 simultaneously with CD3z and other co-costimulatory molecules in one T cell. The overexpression of HER2 in many malignant epithelial cells makes biCAR-T cells expressing HER2 and MUC1 efficient in tumor killing, inducing T cell proliferation, and eradicating antigen escape when encountering target cells. However, investigators found that the activity of engineered T cells was more dependent on the engagement with HER2 regardless of MUC1 interaction and that the CAR-T cells could only be fully activated upon simultaneous expression of two CARs in one cell [66]. In another study, PSCA- and MUC1-targeting CAR-T cells cooperatively killed NSCLC cells expressing PSCA and MUC1 [67]. However, some studies noted that biCAR-T cells are not superior to single CAR-T cells in secreting IL-2 due to the steric hindrance upon simultaneous targeting of both antigens on T cells that prevents the positive feedback on IL-2 [66,68].

Additionally, multi-specific CAR-T cells can be more potent and efficient than unispecific CAR-T cells in improving the durability of CAR response [69]. A biCAR was engineered to combinatorially target two prostate cancer antigens, PSMA and PSCA, although neither antigen alone is exclusively specific to prostate cancer. An intracellular co-stimulatory domain includes a CD3ζ domain and a CD28-4-1BB domain to render this special biCAR effective in eradicating tumors and reducing toxicity against normal tissues positive with a single antigen [70]. Multi-specific CAR-T cells also efficiently limit the “on-target off-tumor” toxicities caused by TAA expression on normal tissues. However, these toxicities are notably challenging to access in preclinical models. Therefore, assumptions regarding clinical safety cannot be extrapolated from preclinical data alone. Preclinical data demonstrated that targeting multiple TAAs minimizes antigen escape variants. Petrov et al. developed CD33-CD123 compound CAR-T cells that target tumor cells expressing both CD33 and CD 123 with impressive anti-leukemic results as it is less likely for tumor cells to lose both antigens [71]. Along with biCARs, structures such as tandem CARs have strengthened the functionality of CAR-T cells by reducing steric interactions with tumor antigens [62].

### 5.2. Induction of Antibody-Dependent Cell Cytotoxicity via the Fc-Gamma Receptor (FcγR)

Another multi-target CAR-T cell approach is Fc-gamma chimeric receptor T cell-based immunotherapy [72,73]. This approach is exemplified by a study in which antibody-dependent cell cytotoxicity (ADCC) competence was accorded to T lymphocytes [74]. ADCC is an important mechanism by which mAbs engage natural killer (NK) cells and other cytotoxic effector cells by recruiting them to the Fc portion of the antibody via the Fc-gamma receptor (FcγR) on the effector cells. This engagement prompts the effector cells to kill targets that include cancer cells and infected cells. ADCC thus bridges innate and adaptive immune responses. Since most T lymphocytes do not have an activating FcγR and do not mediate ADCC, the authors engineered a chimeric receptor composed of FcγR and T-cell–signaling molecules to enable ADCC capability to these cells. The T cells, armed with ADCC capabilities via the Fc receptor FcγR IIIa (CD16) with a valine residue at position 158 (which confers increased Fc binding and tumor cell killing) when combined with the T-cell stimulatory molecule CD3ζ and the co-stimulatory molecule 4–1BB, exhibited sustained proliferation and were cytotoxic to antibody-targeted cancer cells.

Thus, in theory, this approach would enable improved ADCC-associated T-cell cytotoxicity toward any cancer cell expressing tumor-associated antigens (TAAs) that are targetable by therapeutic mAbs. This concept of potential universality of TAA targeting is further illustrated by a study of mogamulizumab (Mog), a humanized mAb that targets a different TAA, namely CC chemokine receptor 4 (CCR4). Mog exhibits ADCC through CD16-expressing effector cells, and a CD16 retroviral vector construct expressing 158V/V-CD3ζ, (cCD16–CD3ζ) was infused in combination with Mog into NOD/scid/γcnull (NOG) female mice along with autologous cCD16ζ-T cells from a patient with aggressive adult T-cell leukemia cells [75]. This therapy was able to suppress disease and prolong survival of the mice. In an earlier analogous study, the CD16-CD3ζ/ FcγR gene stably expressed on the surface of CD4- and CD8-positive cytotoxic T lymphocytes led to ADCC killing of a B-lymphoblastoid cell line targeted with anti-CD20 mAb [76]. Similar successful paradigms that employ CD16-CAR-T cells in the presence of antibody directed against MCSP or CD20 in pancreatic cancer [77] and in the presence of an anti-CD20 mAb directed against immunodeficient mice harboring the CD20-positive Raji lymphoma cell line have been published [78].

In a recent comparison of CD16-CAR-T and CD32-CAR-T cells in KRAS-mutated colorectal carcinoma, even though CD32 was able to effectively bind soluble mAbs and provide a cytotoxic signal, CD32-CAR-T cells did not produce pro-inflammatory cytokines when co-cultured with KRAS-mutated HCT116 colorectal cancer cells, and they had no effect on HCT116 cell viability. However, CD16158V-CAR-T cells combined with cetuximab decreased HCT116 cell viability and SCID mice injected subcutaneously with KRAS-mutated HCT116 cells, followed in one hour by injection of CD16158V-CAR-T cells plus cetuximab near the HCT116 injection site, showed decreased tumor growth and increased disease-free survival [79]. This same group reported that both CD16158F-CAR- T and CD32-CAR-T cells with an A131R polymorphism (CD32A131R-CAR-T cells) were able to induce ADCC toward the triple-negative breast cancer cell line MDA-MB-468, and that the CD32A131R-CAR-T cells were better than CD16158F-CAR-T in redirecting these engineered T cells to MDA-MB-468 cells by way of anti-EGFR mAbs [80].

### 5.3. Tandem CAR-T Cells

Tandem CAR-T (TanCAR) cells express one CAR containing two binding domains linked in tandem, sharing a common intracellular domain. Similar to dual-signaling CARs, TanCAR-T cells expressing two scFv domains on one CAR exert an effect similar to that of dual-signaling CAR-T cells [2]. TanCAR has been proven to kill tumors more efficiently than unispecific CAR by working on a dual-target mechanism. Recognition of one antigen by receptors can trigger an anti-tumor response to offset the escape of the other antigen. TanCAR-T cells engineered for combinatory targeting CD19/CD20 enhance tumor-killing efficiency through sufficient coverage and specificity when recognition of either antigen occurs. These strategies remain a challenge because the choice of validated antigens and suitable epitopes is limited. Grada et al. designed TanCAR using a spacer to connect two different scFv domains in tandem to overcome antigen escape, preserving anti-tumor efficacy when one antigen was downregulated or unexpressed [68]. Additionally, tumor-killing reactivity was enhanced due to the synergistic effect when both antigens simultaneously interacted with their scFv domain in vitro and in vivo [68]. An example is TanCAR-T cells binding either HER2 or IL13Rα2 in glioblastoma cells. TanCARs further increased anti-tumor reactivity and enhanced T cell activation compared to unispecific CAR-T cells when both recognition sites were engaged [81]. In another study of multiple myeloma, Kang et al. confirmed the increased anti-tumor reactivity of TanCAR-T cells against cells expressing CD19 and BCMA relative to CD19- or BCMA-specific CAR-T cells alone [82]. Furthermore, if both antigens are encountered simultaneously, T cell exhaustion and antigen escape can be overcome [81]. However, there is a risk of TanCAR-T cells losing their efficacy over time as the enhanced immune pressure can cause the loss of both antigens due to tumor adaptation, suggesting that more work is needed to design optimal antigen combinations for immunotherapies. Choe et al. recently developed SynNotch T cells expressing TanCAR to enhance targeting precision through the integration of information from multiple imperfect but complementary antigens if targeting individually. Researchers primed synNotch-CAR-T cells through a highly tumor-specific neoantigen such as EGFRvIII, or a tissue-specific antigen such as myelin oligodendrocyte glycoprotein (MOG) in a GBM6 patient-derived xenograft model. synNotch-CAR-T cells showed improved efficacy and durability over individual parental constitutive CAR-T cells while not inducing toxicity or damage to healthy tissue. The α-EGFRvIII synNotch receptor, once primed, induces the expression of tandem α-EphA2/IL13Rα2 CAR for improved specificity and lower T cell exhaustion [83].

### 5.4. ON-Switch CAR-T Cells

Despite promising results in clinical trials, lack of control over-engineered cells once infused into the patient can result in severe adverse events requiring control strategies to improve safety. Excessive cytotoxicity activity and poor control of CAR-T cells have been challenges limiting the application of CAR-T therapies [84,85]. In an in vivo study, a reversible control over CAR-T cell activity was observed using ON-switch CAR-T cells developed to render a titratable regulation to activate the CAR-T cells while mitigating toxicity due to excessive immune response. This approach involved heterodimerized biCARs containing an antigen-binding domain and a co-stimulatory CD3z signaling domain for intracellular signaling. Researchers employed a small molecule dimerizer known as rapamycin analog AP21967 to facilitate a heterodimerized biCAR. CAR-T cells were switched ON upon the dimerization. Therefore, clinicians gained more control of the therapeutic activity of CAR-T cells in terms of dose, timing, and site by changing the dose of a heterodimerizing small molecule [62,86]. Zheng et al. applied this molecule to better control the therapeutic safety profile against glioblastoma. In this study, anti-EGFRvIII synthetic splitting CAR-T cells were activated when EGFRvIII and AP21967 were present. ON-switch CAR-T cells were triggered via the administration of AP21967, which allowed control of the reactivity of synthetic splitting CAR-T cells exogenously in terms of T cell activation, cytokine expression, and tumor cytotoxicity. The switch receptor could efficiently activate CAR-T cells and further enhance cytokine release, proliferation, and anti-tumor reactivity of CAR-T cells [87].

B cells targeting CD19, CD22, and HER2 showed promising results of switchable CAR-T cell potency, demonstrating that clinicians could control the anti-tumor reactivity switch molecule [88,89,90]. In an in vivo study of a xenograft model of breast cancer, anti-HER2 switches were generated by integrating FITC or PNE with a monoclonal antibody against HER2-expressing cells. These FITC or PNE-based switches redirect CAR-T cells so that switchable CAR-T cells can eliminate tumor cells with high selectivity. Conversely, the absence of switch molecules can terminate switchable CAR-T cell activity [89]. In a Nalm-6 xenograft rodent model, anti-CD19 antibody-based switches showed efficiency in redirecting switchable CAR-T cells to clear tumor cells in B cell leukemia and improved safety by mitigating cytokine release syndrome [90]. The more novel approach to generating universal anti-FITC redirected CAR-T cells against CD19- and CD22-expressing cancer cells suggests a broader expansion of switchable CAR-T cells to heterogenous and resistant tumors once monoclonal antibody therapy is of limited efficacy [88,89,90]. Investigators noted that antigen target location and hinge length in switchable CAR-T cells influence anti-tumor effectiveness and can be modified to control timing and dose of CAR-T cell reactivity [89].

### 5.5. Affinity-Tuned CAR

TAAs express with low density in normal cells and T cells targeting TAAs possibly induce on-target off-tissue toxicity. EGFR is overexpressed in tumor cells and has been the target of therapies against various types of cancer. Nevertheless, on-target off-tissue toxicity occurs when normal cells express EGFR at lower levels. Liu et al. generated T cells modified with EGFR-specific CARs to reduce the “on-target off-tissue” toxicity [91]. The novel CAR structures differentiate T cell activation between tumor cells and normal cells based on antigen expression level. Tuning T cell affinity through elevated scFv recognition of antigens can selectively target cells expressing EGFR at high density on glioma cells, while low-density EGFR cells impair functional affinity. These engineered CAR-T cells are promising in other cancers overexpressing EGFR including bladder, cervical, esophageal, head, and neck squamous cell, gastric, breast, ovarian, colorectal, endometrial, and non-small lung cell carcinomas [92,93]. In another strategy, Lynn et al. cloned high-affinity scFv into lentiviral vectors containing CD28-CD3ζ to create a HA-28Z CAR third-generation lentiviral vector for highly sensitive detection of a lower level of antigen FRβ. However, higher affinity does not always mean higher function and sometimes can have the opposite effects. The high-affinity HA-FRβ CAR T-cells reduced the activation threshold and thus potentially diminished “on-target off-tumor” toxicity at low target expression [94].

### 5.6. Inhibitory CAR (iCAR) to Evade Healthy Cells from CAR-T Cell Cytotoxicity

Inhibitory CARs (iCARs) recognize specific antigens expressed on normal or non-cancer cells to direct the CAR-mediated attack away from healthy cells. Thus, induction of the negative signaling keeps CAR-T cells from attacking normal cells. For example, Fedorov et al. evaluated PSMA iCARs, PSMA-targeted CAR-T cells expressing PD-1 and CTLA-4 for negative signaling when encountering PSMA in normal cells [95]. With adenosine as a potential target, CTLA4-target CAR-T cells were engineered to express adenosine A2A receptor (A2AR) to suppress immune response and improve the anti-tumor effect. In this study, iCARs saved healthy cells from the CAR-mediated damage without elimination or irreversible inactivation of CAR-T cells. In other instances, the iCAR strategy enhances antigen recognition and increases safety. In the treatment of B cell malignancies with CD19 CAR-T cells due to the high expression of HLA-C antigen on the surface of healthy B cells in comparison to malignant B cells, CD19-CAR-T cells have exhibited cytotoxicity toward normal B cells expressing CD19. To address this problem, Tao et al. designed an additional iKP CAR on CD19 CAR-T cells to reduce toxicity to CD19-positive normal B cells. iKP CAR, with extracellular killer inhibitory receptors (KIRs) fused with PD-1 through a CD8α hinge and transmembrane domain, can recognize HLA-C on normal cells and exerts a PD-1-based inhibitory effect on the activation of T cells after KIRs have engaged HLA-C. The presence of iKP/CD19 CARs enables T cell differentiation between off-target and on-target B cells to improve the specificity and safety of CAR-T cell therapy [96]. As CD56-positive T cells were more activated and exerted a more potent anti-tumor effect, Zou et al. discovered a novel iCAR simultaneously targeting inhibitory receptors PD-1, Tim-3, and Lag-3, which are known to be overexpressed in tumor-infiltrating lymphocytes (TILs), resulting in the upregulation of CD56 and enhancing the infiltration and anti-tumor function of CAR-T cells [97,98].

Application of suicide switches by inserting suicide genes into CAR-T cells that migrate to tumors and initiate the process of cell suicide can limit the CAR-T cell toxicity [99]. The elimination of endogenous TCRs is another compelling strategy to reduce the side effects of CAR-T cell therapy [100,101]. The suicide gene inducible Casp9 (iCasp9) induces the irreversible depletion of T cells within 30 min after administration [102]. iCasp9 is known to also induce apoptosis of CAR-T cells, thus reducing cytotoxicity toward non-cancer tissues. Additionally, other suicide switches such as herpes simplex thymidine kinase (HSV-TK) and CD20 have already been clinically tested to control the killing activity of CAR-T cells in normal tissue [103,104,105].

### 5.7. Pooled CAR-T Cells

Pooled CAR-T cell therapy involves the infusion of multiple single-targeting CAR-T cells to acquire lower tumor relapse. Each CAR-T cell targets a different cognate antigen. Pooled CAR-T cells secrete a higher level of cytokines and anti-tumor activity than individual CAR-T cells. However, it is of concern that one CAR-T cell may expand over the other as anti-CD19 CAR-T cells are known to increase predominantly over anti-CD20 CAR-T cells, eventually resulting in a net decline of anti-CD20 CAR-T cells. Dual-targeted CAR-T cell therapy is preferred over single-targeted treatment because it prevents CD19-negative recurrence after CAR-T cell treatment. More studies are needed on CD19/CD20, CD19/CD22, or CD33/CD123 dual-targeted CAR-T cells for enhanced CAR-T cell function [106,107]. A phase I study of anti-CD19/CD22 CAR-T for relapsed/refractory NHL is currently being conducted (NCT03448393, ClinicalTrials.gov).

### 5.8. Targeting Tumor Stroma and Vasculature

Cancer cells typically outgrow the vascular system around tumor tissue and require neovascularization for further growth. Angiogenesis and metastasis depend on the expression of vascular endothelial growth factors (VEGF) and their receptors (VEGFRs) in the tumor. Therefore, the unique vascular network around the tissue is a target for CAR-T therapy [108,109]. CAR-T cells designed to target VEGF and VEGFRs have potentiated anti-tumor efficacy and induced tumor regression [108,110]. A highly expressed receptor on malignant endothelial cells, VEGF receptor-2 (VEGFR-2), is a specific target to facilitate T cell infiltration [110,111]. Additionally, anti-angiogenic therapy increases the expression of chemokines and adhesion molecules for more effective infiltration and T cell recruitment [112]. With anti-angiogenic pharmacologic intervention, blocking VEGFR-2 has increased the tumor infiltration of CD8-positive T cells to achieve the long-term therapeutic efficacy of cancer immunotherapy [113].

Targeting other factors in the tumor microenvironment is another therapeutic strategy for CAR-T cell therapy. One example is CAR-T cells constructed against the fibroblast activation protein (FAP) to target cancer-associated fibroblasts (CAPs), which secrete growth factors to the extracellular matrix (ECM) to support tumor growth [114]. FAP-specific CAR-T cells have depleted tumor stroma and reduced vascular density, both of which promote tumor progression and metastasis. Depletion of FAP disrupts the immunosuppressive environment around tumor tissues and relieves therapy resistance. The anti-tumor activity of anti-FAP CAR-T cells includes inhibiting stromagenesis, thus reducing tumor growth by both immune-dependent and immune-independent mechanisms [114,115].

Heparan sulfate proteoglycans (HSPGs) prevent CAR-T cell transport through the ECM in the stroma-rich tumor. Decreased heparanase (HPSE) mRNA expression reduces the anti-tumor function of CAR-T cells in solid tumors because HPSE aids in the penetration of HSPGs, which in turn increases the abundance of the ECM. [116] Therefore, Caruana et al. generated CAR-T cells expressing HPSE to promote T cell infiltration and showed that the improved anti-tumor immunity of CAR-T cells could degrade the stroma and promote T cell infiltration [117].

### 5.9. Directing CAR-T Cells to Chemokine Receptors

The infiltration of CAR-T cells to the tumor site is significantly reduced when the chemokine receptor expressed by T cells is not compatible with chemokines secreted by tumor cells, as noted in the case of CXCL-1, CXCL-5, and CXCL-12. CAR-T cells designed to increase the expression of those chemokine receptors that recognize chemokine secreted by tumors are known to improve the migration of CAR-T cells to a target site. Kershaw et al. developed CAR-T cells expressing chemokine receptor CXCR2 that binds to the ligand CXCL1 on tumor cells. They found that CXCR2 CAR-T cells effectively migrate toward melanoma [118]. In another study, Craddock et al. modified T cells with a CAR specific for tumor antigen GD2 and coexpressing chemokine receptor CCR2b. CCR2b-expressing GD2-CAR-T cells had significantly more tumor-specific trafficking and enhanced inhibitory activity against neuroblastoma xenografts [119]. In other studies, CD30-specific CAR-T cells were designed with CCR4-boosted migratory capacity in murine Hodgkin’s lymphoma xenograft models [120]. Engineered CAR-T cells expressing CC chemokine receptor 2b (CCR2b) showed improved migration in mesothelioma and neuroblastoma cells expressing CC chemokine ligand L2 (CCL2) [120,121]. Furthermore, CXCL-1-receptor CAR-T cells that target the chemokine CXCL-1 derived from melanoma have proven to be effective in directing effector CAR-T cells toward tumor sites [118]. Adachi et al. developed CAR-T cells that simultaneously produce IL-7 and CCL19 with the goal of increasing the recruitment of T cells to tumor sites. The coexpression of IL-7 and CCL19 by CAR-T cells also enhanced the infiltration and survival of CAR-T cells [116]. Conversely, investigators have reported that the absence of suitable chemokine receptors on T cells considerably decreased the migration of CAR-T cells into tumors [122,123]. In both preclinical and clinical trials, studies of locoregional versus intravenous administration of CAR-T cells have been ongoing to overcome the challenge of CAR-T cell trafficking. Preclinical data on the locoregional infusion of CAR-T cells on different solid tumors have shown superior efficacy over systematic distribution on primary or secondary pleural malignancies, peritoneal tumors, multifocal brain metastasis, relapsed medulloblastoma, central nervous system lymphoma, and ependymoma [124,125,126,127,128,129]. Most notably, Adusumilli et al. demonstrated that intrapleural administration of MSLN-targeted CAR T cells in patients with primary or secondary pleural malignancies maintained tumor eradication and functional T cells for up to 200 days. Similarly, Mulazzani et al. compared intracerebral versus intravascular injection of anti-CD19 CAR-T cells in animal models of primary central nervous system (CNS) lymphoma. Intracerebral delivery allowed deeper filtration to tumor sites and promoted vascular system and intracerebral and intravascular persistence for up to 159 days after injection, thus prolonging anti-tumor activity and animal survival [128]. A Phase I clinical trial by Katz et al. stated that intraperitoneal delivery of CAR-Ts for peritoneal carcinomatosis provided superior anti-tumor activity and further demonstrated that combined CAR-T treatment with depleting antibodies against myeloid-derived suppressor cells and Treg, found with high frequency at peritoneal tumors, further improved efficacy against peritoneal metastases [125]. In a recent Phase 1 clinical trial of HER2-specific CAR-T cells on pediatric and young adult glioblastoma patients, Vitanza et al. studied locoregional diffusion to the tumor cavity or ventricular system and showed local activation of CNS immune response without dose-limiting toxicity [127,130]. In summary, with impressive tumor eradication, limiting systemic distribution, and without detectable systematic toxicity [126], locoregional distribution of CAR-T cells might be clinically the most effective route of administration while maintaining safety in the setting of solid tumors.

## 6. Conclusions

In the last decade, CAR-T immunotherapy has become an exciting approach with great potential in precision medicine. Novel strategies have been developed to enhance the tumor-killing activity, endurance, infiltration to solid tumor tissues, and regulation of the immune microenvironment. Combined therapies are being developed to improve immune response, minimize “on-target off-tumor” effects, and transform immunologically “cold” to “hot” tumors. More studies are focused on identifying ideal target selection and targeting multiple tumor-associated antigens. Equally important, development in optimal treatment timing, persistence of CAR-T cells, and expansion of the CAR-T cells within the tumor immunosuppressive microenvironment have been further addressed. The configuration of these therapies will result in more effective treatment and improved response in tumors.

## Data Availability

No new data were created or analyzed in this study. Data sharing is not applicable to this article.
